# Supplementary material for: The roles of MRI-based prostate volume and associated zone-adjusted prostate-specific antigen concentrations in predicting prostate cancer and high-risk prostate cancer
Source: PLoS One. 2019 Nov 19;14(11):e0218645. doi: 10.1371/journal.pone.0218645 (PMC6863612; doi:10.1371/journal.pone.0218645)
Supplement: Attachment — Submitted filename: Response to Reviewers.doc [file pone.0218645.s002.doc]

Dear Editors and Reviewers:

Thank you for your letter and for the reviewers’ comments concerning our manuscript entitled “The roles of MRI-based prostate volume and associated zone-adjusted prostate specific antigen concentrations in predicting prostate cancer and high-risk prostate cancer” (Manuscript number: PONE-D-19-16015). Those comments are all valuable and very helpful for revising and improving our paper, as well as the important guiding significance to our researches. We have studied comments carefully and have made correction which we hope meet with approval. Revised portion are highlighted in a marked-up copy of my manuscript. The main corrections in the paper and the responds to the reviewer’s comments are as following:

Responds to the reviewer’s comments:

Reviewer #1:

1. Please elaborate on the 13 core biopsy technique you used. I am more familiar with a 12 core sampling of the peripheral zone. Some urologists also perform anterior biopsies for a total of 14 cores. Targeted biopsies based on multiparametric MRI findings are then done.

Response: Thank you for suggesting us to make a more clear statement of the 13-core biopsy technique that we used in our clinical work. As we mentioned in our manuscript, four and two cores were acquired from the left PZ and left TZ, respectively, and four and two cores were acquired from the right PZ and right TZ, respectively. The last core was examined for abnormalities found in imageological examination. The number of biopsy cores still remains inconclusive. In our routine clinical work, we perform standard TRUS-guided 13-core prostate biopsy for patient.

2. Were your targeted biopsies "cognitive" or did you use MRI-U/S fusion technology such as Artemis or UroNav?

Response: We are sorry for our negligence of the method of our targeted biopsies. We did use cognitive fusion technology in the last core of biopsy.

3. What percentage of standard biopsies were positive for cancer vs. those targeting a MRI lesion?

Response: Thank you for putting forward such a valuable question. The last core, so called cognitive biopsy, was a part of systematic biopsies. We didn’t compare the positve rate of each core.

4. Please format the references according to journal specifications. Numerous citations were missing author names.

Response: As Reviewer suggested, our software had something wrong in citing references. But we have fixed it and made correction according to Endnote style file, named “PLoS (Public Library of Science – all journals)”, downloaded from PLOS ONE.

Special thanks to you for your good comments.

Reviewer #2:

1. Please clarify - Ethics Statement on Page 3 is “N/A” and the “Materials and Methods” states “Ethics approval was acquired from the IRB…” Please adjust the Ethics statement accordingly.

Response: It is really true as Reviewer suggested that we missed the ethics approval statement. Ethical approval was acquired from the Institutional Review Board of Fujian Medical University Union Hospital. The approval form of consent was obtained by written with approval number of 2018KY078. Now we have submit the ethics approval statement in submission system.

2. Please clarify: “Material and Methods” states this was “retrospective cohort study” and the “Clinical Date and Variable Definition” states ALP and LDH were collected before biopsy. Was that part of the study or was it standard of care. This is important due to the concern above regarding ethics statement.

Response: Thank you for indicating the potential ethics problem. We routinely perform biochemical analysis on patients, which including ALP and LDH. So it is standard of care for patients.

3. If you feel appropriate, I would like your thoughts on the existing PIRADs classification used in clinical practice and how it relates to your findings.

Response: Thank you for giving us a hint that will provide a good perspective for improving clinical practice. PIRADs classification is a well-recognized technique that can improve diagnostic performance in prostate cancer. Now we are still working on the PI-RADs v2. And I believe we will get acquainted with it in our next research.

Special thanks to you for your good comments.

Responds to editor comments:

1.We have ensured that our manuscript meets PLOS ONE's style requirements through PLOS ONE style templates. If our manuscript still exists any problem, please tell me.

2.The information of patients which we used in our retrospective study has been mentioned in the Clinical data and variable definitions part in our manuscript. We have ensured that all data were fully anonymized before we accessed them.

3.Considering the words limitation of abstract, so we didn’t define abbreviations in the abstract. But as editor suggested, now we have defined abbreviations in the abstract, including PCa, PV, PSAD, TZV, PSADTZ, PZV and PSADPZ.

We appreciate for Editors/Reviewers’ warm work earnestly, and hope that the correction will meet with approval.

Once again, thank you very much for your comments and suggestions.

Sincerely,

Mengqiang Li
